# Predictors of adherence to a 12‐week exercise program among men treated for prostate cancer: ENGAGE study

**DOI:** 10.1002/cam4.639

**Published:** 2016-02-12

**Authors:** Melinda Craike, Cadeyrn J. Gaskin, Kerry S. Courneya, Steve F. Fraser, Jo Salmon, Patrick J. Owen, Suzanne Broadbent, Patricia M. Livingston

**Affiliations:** ^1^ Deakin University GeelongSchool of PsychologyCentre for Social and Early Emotional Development Faculty of HealthGeelongAustralia; ^2^ Behavioral Medicine LaboratoryFaculty of Physical Education and RecreationUniversity of AlbertaEdmontonCanada; ^3^ Deakin University GeelongSchool of Exercise and Nutrition Sciences, Centre for Physical Activity and Nutrition Research (C‐PAN)Faculty of HealthGeelongAustralia; ^4^ School of Health and Human SciencesSouthern Cross UniversityLismoreAustralia; ^5^ Deakin University GeelongFaculty of HealthGeelongAustralia

**Keywords:** Adherence, exercise, neoplasms, physical activity, prostatic neoplasms

## Abstract

Understanding the factors that influence adherence to exercise programs is necessary to develop effective interventions for people with cancer. We examined the predictors of adherence to a supervised exercise program for participants in the ENGAGE study – a cluster randomized controlled trial that assessed the efficacy of a clinician‐referred 12‐week exercise program among men treated for prostate cancer. Demographic, clinical, behavioral, and psychosocial data from 52 participants in the intervention group were collected at baseline through self‐report and medical records. Adherence to the supervised exercise program was assessed through objective attendance records. Adherence to the supervised exercise program was 80.3%. In the univariate analyses, cancer‐specific quality of life subscales (role functioning *r *=* *0.37, *P *=* *0.01; sexual activity *r *=* *0.26, *P *=* *0.06; fatigue *r *=* *−0.26, *P *=* *0.06, and hormonal symptoms *r *=* *−0.31, *P *=* *0.03) and education (*d* = −0.60, *P *=* *0.011) were associated with adherence. In the subsequent multivariate analysis, role functioning (B = 0.309, *P *=* *0.019) and hormonal symptoms (B = −0.483, *P *=* *0.054) independently predicted adherence. Men who experienced more severe hormonal symptoms had lower levels of adherence to the exercise program. Those who experienced more positive perceptions of their ability to perform daily tasks and leisure activities had higher levels of adherence to the exercise program. Hormonal symptoms and role functioning need to be considered when conducting exercise programs for men who have been treated for prostate cancer.

## Introduction

Worldwide, prostate cancer is the second most frequently diagnosed cancer in men; in 2012, an estimated 1.1 million new cases were diagnosed, accounting for 15% of the cancers diagnosed in men 1. With earlier detection and effective treatments, more men with prostate cancer are living longer postdiagnosis; five‐year relative survival rates in Australia increased to 93.0% for the years 2007–2011 2. Given the high survival rates for prostate cancer, it is important that survivorship issues are addressed.

Men who have been treated for prostate cancer often experience high rates of morbidity due to ongoing adverse physical effects of treatment, decision‐related distress, and the psychological effects of living with cancer 3, 4, 5. Thus, improving the quality of life and psychological well‐being of these men through supportive care interventions such as exercise programs is a priority 6. Exercise has a range of benefits for men who have been treated for prostate cancer and evidence supports the positive physical and psychological outcomes of exercise for this group 6, 7, 8, 9. Recommendations for physical activity for cancer survivors include at least 150‐min per week of moderate intensity or 75‐min per week of vigorous‐intensity aerobic physical activity, or an equivalent combination. Two to three weekly sessions of resistance training are also recommended 10 Most men who have been treated for prostate cancer, however, do not achieve recommended levels of physical activity 11, 12, in a recent study only 12.3% reported sufficient levels 12. Lifestyle interventions that focus on increasing adherence to exercise can help to address low levels of participation.

Examination of factors that are associated with adherence to exercise interventions for people with cancer will assist in the tailoring of these programs and potentially lead to higher levels of adherence and reduced attrition 13, 14. However, researchers rarely investigate predictors of adherence to exercise. 13, 14, 15. Given that adherence rates and predictors differ across cancer groups and types of exercise intervention (supervised or home‐based or combination) 13, 16, 17, the examination adherence for specific cancer groups and types of interventions is required 14. To our knowledge, only one study has reported predictors of adherence to an exercise program for men treated for prostate cancer and was limited to men receiving androgen deprivation therapy (ADT) 18. In that study, Courneya et al. reported that three factors independently explained 20.4% of the variance in adherence: exercise stage of change, age, and intention.

This study is a secondary analysis of data from the ENGAGE study. The aims of the ENGAGE study were to determine the efficacy of a clinician referral and exercise program to improve exercise levels and quality of life for men with prostate cancer and the main outcomes of the study have been reported previously 19. Briefly, we found a significant positive intervention effect for vigorous‐intensity exercise; meeting exercise guidelines; improved cognitive functioning and a reduction in symptoms of depression 19. The goal of this secondary analysis was to examine demographic, clinical, behavioral, and psychosocial predictors of adherence to the supervised exercise program. Given the lack of research into the factors that predict adherence to exercise interventions for men treated for prostate cancer, we undertook an exploratory approach that included a range of potential predictors.

## Method

Details of the ENGAGE study methods have been reported elsewhere 20 and are briefly presented here. The study was a two‐armed prospective, multicenter, cluster randomized controlled trial. Ethical approval was obtained from Deakin University and each of the health services involved in the study. Written informed consent was obtained from patients prior to their involvement in the study.

### Participants and procedure

Participants in this study were adult men who had (a) a diagnosis of stage I, II, or III prostate cancer and treated with curative intent; (b) completed treatment for prostate cancer within the previous 3–12 months (patients on ADT were eligible to participate); and (c) the ability to complete surveys in the English language. Participants were excluded if they had any musculoskeletal, cardiovascular, or neurological disorders deemed absolute contraindications to exercise.

Recruitment was conducted at urology and radiation oncology outpatients’ clinics within three public health services and four private clinics in Melbourne, Australia. Patients were identified through health services and private clinic patient records and recruited from October 2011 to June 2013. Patients were informed about the study and provided with an information package at their clinical consultations. Clinicians randomized to the intervention condition provided a referral slip to patients deemed suitable and recommended that the patient undertake the exercise program. Clinicians randomized to the control condition provided usual care regarding physical activity advice. Clinicians provided medical clearance prior to the patients’ involvement in the study. A study researcher later phoned eligible patients to explain participation in the study in more detail, answer any questions and gain informed consent.

### Randomization

Clinicians (i.e., urologists, radiation oncologists, and urology nurses) who agreed to be involved in the study were randomly allocated using an online random number generator to either the intervention or control conditions. Randomization was conducted by the first author.

### Exercise training intervention

We specifically designed the 12‐week exercise intervention to address the determinants of social cognitive theory (SCT) 21, 22 and aimed to increase physical activity participation, based on physical activity guidelines for cancer survivors proposed by the American College of Sport Medicine 10 and Exercise Sport Science Australia 23. Participants were asked to complete three, up to 50 min, sessions of exercise training per week. Two of these sessions were supervised and conducted with exercise trainers who were postgraduate clinical exercise physiology students under the supervision of accredited exercise physiologists (a tertiary‐trained exercise training professional) and one session was a nonsupervised home‐based session. Exercise sessions incorporated progressive aerobic, resistance, balance, and flexibility exercises and was tailored to the needs and ability of participants. Each supervised session included 20–30 min of aerobic exercises prescribed at 40–70% of maximum heart rate and 4–6 upper and lower body resistance exercises, performed in two sets of 8–12 repetitions. To achieve consistency in exercise prescription, each exercise program included 90° leg press, seated chest press, and seated row.

Exercise trainers received training in SCT and were instructed to incorporate discussions of the participant's beliefs in their ability to exercise, exercise preferences, outcome expectations, goals, and strategies for using facilitators and overcoming barriers to performing exercise. Exercise trainers were provided with checklists to ensure that they were addressing the key topics during exercise sessions. The involvement of clinicians in referring men to the program provided social support, which was expected to increase self‐efficacy through increasing men's beliefs in their ability to undertake exercise.

### Measures

#### Outcome variable

The outcome for the secondary analysis was *Adherence to the Exercise Program*, assessed as the percentage of sessions that were attended in the supervised component of the exercise program. The percentage of sessions was calculated as follows: the number of sessions attended/24 planned sessions × 100. The number of sessions attended was assessed by objective attendance records maintained by the exercise trainers in the study. We used an intention to treat analysis.

#### Predictor variables

Previously validated scales were used to measure cancer‐specific quality of life, depressive symptoms, and prostate cancer‐related anxiety. *Cancer‐specific quality of life* was measured using The European Organization for Research and Treatment of Cancer core quality of life questionnaire (EORTC QLQ‐C30, V3) 24 and the prostate tumor‐specific module (EORTC QLQ‐PR25) 25, 26; *Depressive symptoms* were measured using the 20‐item Centre for Epidemiological Studies Depression Inventory (CES‐D) 27; *Prostate‐cancer‐related anxiety* was measured using the Memorial Anxiety Scale for Prostate Cancer (MAX‐PC) 28.

An adapted version of the Leisure Time Exercise Questionnaire 29, 30, was used to measure *past physical activity behavior*. Participants were asked to report their average weekly duration of light (minimal effort, no perspiration), moderate‐ (not exhausting, light perspiration), and vigorous‐intensity (heart beats rapidly, sweating) activity in a typical week in the past month, in addition to the frequency. We then calculated the percentage of participants achieving ≥150 min per week of moderate plus vigorous‐intensity physical activity.

*Demographic characteristics* included age, highest level of education, marital status, and body mass index (BMI). BMI was calculated from objective weight (kg)/height (m)^2^. *Clinical characteristics* that were collected included cancer stage and number of weeks since treatment completion, which were collected from medical records, and treatment type (surgery, radiotherapy and/or ADT) and comorbidities (e.g., high blood pressure, high cholesterol, heart disease, arthritis, diabetes, asthma/emphysema, chronic pain), which were collected through self‐report. Total comorbidities were calculated based on the number of comorbidities reported.

### Data analysis

Two of the 54 intervention participants were excluded from this analysis due to insufficient data. Data from 52 participants were analyzed. Overall, missing data for variables included in this analysis were less than 1%. The amount of missing data that was tolerated and scale calculations were consistent with recommendations for the EORTC QLQ‐30 31, EORTC QLQ‐PR25 26, and CES‐D 27. The MAX‐PC was calculated if more than 80% of items were answered. The cancer‐specific quality of life subscale ‘Nausea and Vomiting’ was not included in the analysis as almost all participants (96.2%) reported that they did not experience this symptom. Data for the demographic and clinical characteristics were included in the analysis when data were available for that particular participant.

Our main outcome variable was adherence to the structured exercise training program, expressed as the percentage of total sessions. Data were somewhat non‐normal, however, we made a decision not to transform the data as the tests that we used were robust against non‐normality 32, 33.

The following variables were dichotomized prior to the analyses: highest level of education (categorized as less than university degree or university degree or higher), relationship status (categorized as partner or no partner), ADT (yes/no), and most recent active treatment (radiotherapy or surgery).

First, univariate analysis was conducted to examine associations between the predictors and adherence using *t*‐tests, and Pearson's correlations, where appropriate. Cohen's *d* effect sizes were calculated for *t*‐test statistics. Due to our small sample size, variables that had statistically significant or borderline significance (*P *<* *0.10) univariate associations with adherence were included in the multivariate analysis to examine the independent predictors of adherence to the exercise program.

The clustering effect of clinicians for adherence to the exercise program was moderate (ICC 0.126). We used Generalized Estimation Equation Modelling (GEE) with exchangeable variance‐covariance matrix to adjust for the clinician clustering effect. Backwards deletion method was used to define the final GEE model. This involved deletion of each of the nonsignificant predictors (starting with the largest *P* value) one at a time until significant variables remained.

SPSS (V22, Armonk, NY, IBM Corp.) was used for the descriptive and univariate analyses. STATA (V13, College Station, TX, StataCorp LP.) was used for the GEE analyses.

## Results

The flow of participants has been summarized elsewhere 19. Briefly, 15 clinicians (71.0%) agreed to participate (8 clinicians were randomized to the intervention condition and seven were randomized to the control condition). In total, 320 eligible patients were approached to participate in the study (142 intervention; 178 control), of these, 147 (46%) consented to be involved in the study 19.

The 52 intervention participants included in this analysis ranged in age from 50 to 84 years (M* *=* *67.3, SD = 8.08). Most had completed a certificate or diploma (*n *=* *18, 34.6%) or university degree (*n *=* *16, 30.8%) and were married or living with a partner (*n *=* *45, 86.5%). Their BMI ranged from 19.2 to 36.4 (M* *=* *28.6, SD = 3.74, *n *=* *51). Overall, 16 (30.8%) were treated with surgery only; 15 (28.8%) were treated with surgery and radiotherapy; 13 (25.0%) were treated with radiotherapy and ADT. The mean time since completion of last treatment was 25.5 weeks (SD = 11.37). Most participants were cancer stage I (*n *=* *19, 47.5%).

Scores on the CESD scale ranged from 0 to 45 (M* *=* *9.0, SD = 9.40) and scores on the MAX‐PC ranged from 0 to 36 (M* *=* *9.1, SD = 8.40). In terms of cancer‐specific quality of life, participants reported relatively high functional scores and low symptomology. At baseline, 36.5% (*n* = 19) were participating in ≥150 min per week of moderate plus vigorous physical activity.

Based on the percentage of sessions attended, mean adherence to the structured exercise training program was 80.3% (SD* *= 20.145, *n *=* *52). Attrition was 13.0% (*n* = 7); four participants dropped out or withdrew from the program (7.4%) and three were lost to follow‐up (5.5%).

Participants were asked to record exercise details in an exercise diary for the home‐based component of the program. Among the participants who returned the diary (*n *=* *40) and based on the percentage of sessions that participants reported, the mean adherence was 77.5% (SD = 28.26). Given that 26% of participants did not return their diary, we did not have a full dataset to examine the predictors of adherence to the home‐based component of the program. The association between adherence to the home‐based program and adherence to the supervised program was low to moderate in strength, but not statistically significant (*r *=* *0.27, *P* = 0.096).

The results of the univariate analyses are presented in Tables 1 and 2. Factors that were significantly associated with adherence to the exercise program were as follows: cancer‐specific quality of life factors: role functioning (*r *=* *0.37 *P *=* *0.01, *n *=* *52), sexual activity (*r *=* *0.26, *P *=* *0.06, *n *=* *52), fatigue (*r *=* *−0.26, *P *=* *0.06, *n *=* *52), hormonal symptoms (*r *=* *−0.31, *P *=* *0.03, *n *=* *52), and level of education (*t *=* *−2.65, *P *=* *0.01, *n *=* *52),.

**Table 1:** Behavior and psychosocial factors associations with adherence: univariate analyses

	*n*	*r*	*P*
Memorial Anxiety Scale	52	−0.14	0.34
CES‐D	52	−0.05	0.71
QLQ‐C30			
Global quality of life	52	0.14	0.32
Functional Scales			
Physical functioning	52	0.23	0.10
Role functioning	52	0.37	0.01
Emotional functioning	52	0.19	0.17
Cognitive functioning	52	0.14	0.32
Social Functioning	52	0.06	0.65
Symptom Scales/Items			
Fatigue	52	−0.26	0.06
Pain	52	0.05	0.72
Dyspnoea	52	0.06	0.66
Financial difficulties	51	−0.16	0.26
Diarrhoea	51	−0.17	0.24
Constipation	51	0.00	0.98
Appetite loss	52	0.15	0.31
Insomnia	52	0.07	0.63
QLQ‐PR25			
Functional scales			
Sexual activity	52	0.26	0.06
Sexual functioning^a^	19	0.15	0.55
Symptom scales/items			
Urinary symptoms	52	0.14	0.32
Bowel symptoms	52	−0.08	0.59
Hormonal symptoms	52	−0.31	0.03
Incontinence aid^b^	16	0.07	0.80
	*n*	*t*	*P*
Physical Activity			
≥150 min per week^c^	52	−.585	0.56

a Items were conditional on being sexually active.

b Item conditional on requiring an incontinence aid.

c Cohen's *d *=* *−0.172.

**Table 2:** Demographic and clinical characteristics associations with adherence: univariate analyses

	*n*	***r***	*P*
Comorbidities	49	−0.08	0.58
Age	52	−0.01	0.92
Body mass index (BMI)	51	−0.11	0.45
Weeks since treatment completion	52	−0.10	0.48
Cancer Stage	40	0.088	0.59
	*n*	*t*	*P*
Relationship status	52	0.32	0.75^a^
Surgery or radiotherapy	52	−1.62	0.11^b^
ADT treatment	52	1.62	0.12^c^
Education	52	−2.65	0.01^d^

Cohen's *d* effect sizes:

a relationship status: 0.130;

b Surgery or radiotherapy −0.497;

c Androgen deprivation therapy (ADT) treatment = 0.614;

d education = −0.60.

Significant univariate factors were entered as a single block into a GEE model. None of the individual variables significantly predicted adherence. Following a process of backward variable selection, hormonal symptoms (B = −0.483, *P *=* *0.054, 95% CI = −0.976 to .009) and role functioning (B = 0.309, *P *=* *0.019; 95% CI = 0.051–0.568) were the only significant predictors of adherence (Table 3). Findings suggested that a 10‐point increase in role functioning (scale range 0–100) was associated with a 3% increase in adherence and a 10‐point increase in hormonal symptoms (scale range 0–100) was associated with a 5% decrease in adherence.

**Table 3:** Gee model of quality of life factors on adherence to the exercise program

				95% CI	
	B	z	*P*	Lower	Upper
Model					
Role functioning	0.309	2.34	0.019	0.051	0.568
Hormonal symptoms	−0.483	−1.92	0.054	−.976	.009

## Discussion

We examined the predictors of adherence to a supervised exercise program for men treated for prostate cancer enrolled in a multicenter cluster randomized controlled trial (RCT) and addressed a significant shortcoming in the literature 13, 14. We examined a range of potential predictors of exercise program adherence, including demographic, clinical, behavioral, and psychosocial factors. Level of education and several cancer‐specific quality of life factors predicted adherence to the exercise program in univariate analyses, in subsequent multivariate analyses, role functioning and hormonal symptoms independently predicted adherence to the program.

A novel finding of our study was that men who experienced more severe hormonal symptoms had lower adherence to the exercise program. Men who experience hormonal symptoms, which are adverse effects of ADT 5, may require additional physical and emotional support and assistance to facilitate their ongoing participation in exercise programs. For example, flexibility in the delivery and modifying the intensity of the exercise program when symptoms are present. Although our finding on the effect of hormonal symptoms in men with prostate cancer is new, Courneya et al. 34 recently found that hormonal symptoms were negative predictors of adherence to a supervised exercise program for women undergoing chemotherapy treatment for breast cancer. The influence of hormonal symptoms on exercise participation warrants further investigation; it is possible that the severity of adverse effects from ADT is a more salient factor than the existence of the treatment itself in reducing adherence to exercise training. Furthermore, adverse effects of ADT may exacerbate the impact of other forms of treatment on quality of life 35.

We also found that men who experienced more positive role functioning had higher levels of adherence to the exercise program. Role functioning refers to the patient's ability to pursue activities such as work or other leisure activities and therefore it is not surprising that those who had higher level of functioning in this area would also be more able to adhere to an exercise program. It is possible that experiencing fatigue and hormonal symptoms might reduce the patient's ability to participate in daily activities, therefore influencing participation in an exercise program.

Fatigue, sexual activity, and level of education were significantly associated with adherence in univariate analyses, but not in the subsequent multivariate analysis. This result was similar to a previous study of men treated for prostate cancer, which showed that prostate cancer‐specific quality of life and fatigue predicted adherence to a structured exercise program in univariate analyses, although these factors were not significant in subsequent multivariate analysis 18. Fatigue is regularly experienced by cancer patients 36 and has been cited as a barrier to physical activity for people living with cancer, including men with prostate cancer 37. In our study, men who had completed a University degree achieved a higher level of adherence to the program. Although level of education has rarely been identified as a predictor of adherence to supervised exercise programs in cancer survivors, a study of women with breast cancer undergoing chemotherapy found that education significantly predicted adherence 38. Furthermore, studies of noncancer populations consistently show that higher levels of education are associated positively with participation in physical activity 39, 40, 41. People with higher levels of education may be more informed of the health consequences of certain lifestyle behaviors, leading them to exercise more often 41. The influence of education is likely to be more pronounced in older age because people with low education tend to rely on employment as a major source of physical activity, which results in declines in physical activity as they move through early old age and transition out of the workforce 42.

It is worth noting that, in univariate analyses, several factors that were not statistically significance had medium effect sizes. This suggests that we did not have enough power to detect significance. The factors with medium effect sizes included cancer‐specific quality of life, physical functioning (*r *=* *0.23); ADT (*d* = 0.614; men treated with ADT had lower adherence), and most recent active treatment (*d* = −0.497; men whose most recent treatment was radiotherapy had lower adherence). Consideration should be given to these factors in studies with larger sample sizes.

There is little consensus with regard to factors that are associated with adherence to supervised exercise programs and in many studies, including ours, only a modest amount of the variance in adherence is explained by patient‐related factors 38, 43, 44. This might be due to external factors, such as the nature of supervised exercise programs, which typically have a high level of patient support and features that facilitate adherence and minimize drop out. The factors inherent in supervised exercise programs are likely to result in high adherence and may ameliorate the potential influence of patient related factors in program adherence 17, 44. Based on SCT, our study incorporated behavior change techniques, including a clinician referral, setting program goals, prompting practice and self‐monitoring. A recent review of the literature suggested that such practices were related to higher levels of adherence to exercise programs for people with cancer 45. Thus, incorporating behavior change techniques may also explain the high levels of adherence to the exercise program.

We did not assess predictors of adherence to the home‐based program as 26% of participants did not complete and return their physical activity diary. We did, however, examine the association between participation in the home‐based sessions and supervised sessions and this association was low to moderate in strength and not significant. It is difficult to speculate on whether different factors would have predicted adherence to the home‐based program compared to the supervised program. The patient‐related factors that we examined, however, may have been stronger predictors of the home‐based program because these unsupervised sessions did not have the structure and social support inherent in the supervised program.

This study was strengthened by the use of objective measures of exercise session adherence and inclusion of a range of potential factors that might influence adherence. A limitation of our study was the sample size. Although comparable to similar studies of supervised exercise programs 17, 43, 44, our sample size was relatively small in terms of detecting statistical significance in predicting adherence.

In summary, we suggest that factors related to cancer‐specific quality of life may predict adherence to supervised exercise programs for men treated for prostate cancer. The quality of life of men entering exercise programs should be assessed so that symptoms, including fatigue and hormonal symptoms that impact on their ability to perform daily activities, can be addressed. We also suggest that it would be useful to examine factors external to the patient, including the nature of the exercise program, to further develop our understanding of adherence to supervised exercise programs.

## Conflicts of Interest

None declared.
